# Proteomic Profiling of Dilated Cardiomyopathy Plasma Samples — Searching for Biomarkers with Potential to Predict the Outcome of Therapy

**DOI:** 10.1021/acs.jproteome.3c00691

**Published:** 2024-02-16

**Authors:** Jana Klimentova, Pavel Rehulka, Jiri Stulik, Vera Vozandychova, Helena Rehulkova, Ivana Jurcova, Marie Lazarova, Renata Aiglova, Jiri Dokoupil, Juraj Hrecko, Radek Pudil

**Affiliations:** † Faculty of Military Health Sciences, Department of Molecular Pathology and Biology, University of Defence, Trebesska 1575, Hradec Kralove 50001, Czech Republic; ‡ The first Department of Internal Medicine − Cardioangiology, Medical Faculty of Charles University in Hradec Kralove and University Hospital Hradec Kralove, Sokolska 581, Hradec Kralove 50005, Czech Republic; § Charles University, Faculty of Medicine in Hradec Kralove, Simkova 870, Hradec Kralove 50003, Czech Republic; ∥ Institute for Clinical and Experimental Medicine (IKEM), Videnska 1958/9, Prague 14021, Czech Republic; ⊥ Department of Internal Medicine I − Cardiology, Faculty of Medicine and Dentistry, Palacky University and University Hospital Olomouc, Zdravotniku 248/7, Olomouc 77900, Czech Republic

**Keywords:** dilated cardiomyopathy, left ventricular reverse remodeling, proteomics, plasma proteome profiling, LFQ, functional enrichment analysis

## Abstract

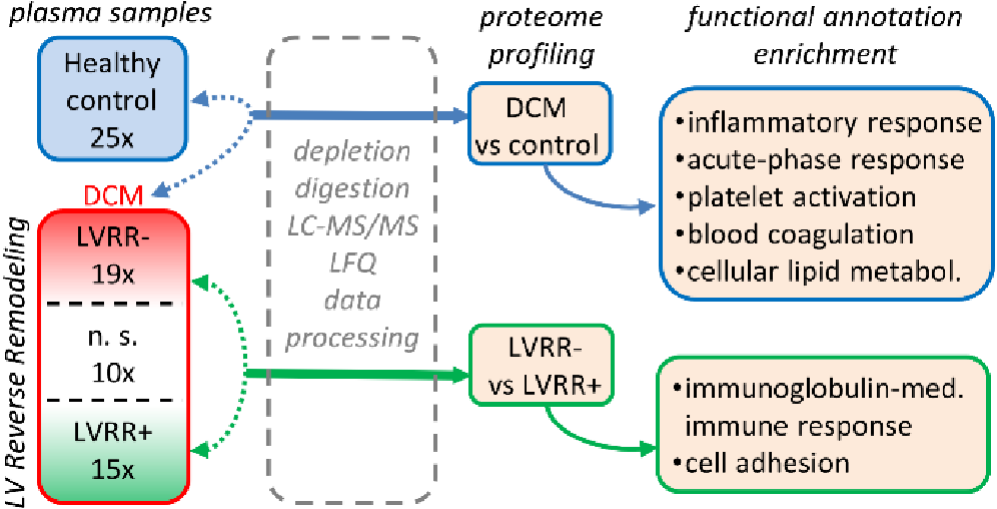

Determination of the prognosis and treatment outcomes of dilated cardiomyopathy is a serious problem due to the lack of valid specific protein markers. Using in-depth proteome discovery analysis, we compared 49 plasma samples from patients suffering from dilated cardiomyopathy with plasma samples from their healthy counterparts. In total, we identified 97 proteins exhibiting statistically significant dysregulation in diseased plasma samples. The functional enrichment analysis of differentially expressed proteins uncovered dysregulation in biological processes like inflammatory response, wound healing, complement cascade, blood coagulation, and lipid metabolism in dilated cardiomyopathy patients. The same proteome approach was employed in order to find protein markers whose expression differs between the patients well-responding to therapy and nonresponders. In this case, 45 plasma proteins revealed statistically significant different expression between these two groups. Of them, fructose-1,6-bisphosphate aldolase seems to be a promising biomarker candidate because it accumulates in plasma samples obtained from patients with insufficient treatment response and with worse or fatal outcome. Data are available via ProteomeXchange with the identifier PXD046288.

## Introduction

Dilated cardiomyopathy (DCM) is a disease of the heart muscle defined by the presence of left ventricular dilatation and global or regional systolic dysfunction unexplained solely by abnormal loading conditions (e.g., hypertension, valve disease, coronary heart disease) or coronary artery disease.^1−3^ It presents one of the most common causes of heart failure worldwide, with an estimated prevalence of 0.036–0.400%,^3^ and it is also the most common indication for heart transplantation.^4^ In the USA, it accounts for ca. 50 000 hospitalizations and nearly 10 000 deaths each year.^5^ DCM has multiple etiologies—genetic and nongenetic.^1^ Genetic causes account for 30–40% of DCMs and involves genes that encode cytoskeletal, sarcomere, and nuclear envelope proteins among others. Acquired causes include myocarditis, tachyarrhythmia, alcohol abuse, drugs, catechol amines, toxins, and metabolic or endocrine disturbances.^4^

During the last decades, the 10-year survival free from heart transplantation has improved impressively, and currently it is close to 85%.^6^ Nevertheless, the outcome of patients with recent onset DCM often remains unpredictable, and major adverse events may occur in the first months following the diagnosis.^2,7^ The socioeconomic impact of adverse events is amplified by the fact that DCM often affects patients in the first decades of life. The management of DCM is generally directed toward major clinical manifestations of heart failure and arrhythmias and includes pharmacological treatment, electrical device therapies, mechanical support, and heart transplantations.^4^ Enhancement in the left ventricular ejection fraction (LVEF) has been considered one of the most important determinants of the improvement in the prognosis of DCM. Left ventricular reverse remodeling (LVRR) is defined as an improvement in LVEF and a reduction in left ventricular dimension.^8^ Reverse remodeling can take place spontaneously upon removal of the inciting cardiac insult (e.g., in tachycardia-induced or toxin-induced cardiomyopathy), but it is, more often, the result of evidence-based pharmacological and nonpharmacological therapies.

The prediction of improvement of LVEF at the initial diagnosis of DCM is of high prognostic significance. The predictors of this process have been intensively studied, but up to now the conventional techniques—echocardiography, biomarker studies, radionuclide ventriculography, and cardiac magnetic resonance—have not provided any breakthrough in the early prediction of improvement of myocardial function.^9−11^ Current proteomic and bioinformatic methods allow for the deep characterization of disease-specific protein markers from tissues or body fluid samples. In DCM, a number of biomarkers have been explored,^12,13^ yet only a few of them are commonly used in clinical practice.^14^ Beyond individual biomarker discovery, the proteomic analyses improved current knowledge about the molecular mechanisms of idiopathic DCM pathogenesis. The dysregulation in major biological processes of the immunology and inflammatory response, lipid metabolism or blood coagulation has been reported in DCM patients.^13^

In the current study, we report a comprehensive proteomics analysis of DCM patients’ plasma samples to recognize specific proteome profile of DCM in comparison to healthy control. In the second part of the study, we focused on the fate of the DCM patients one year after the diagnosis of DCM. In this part, we divided the patients according to their treatment response into subgroups of those whose LVEF improved and those with poor or fatal outcome. We employed mass spectrometry together with the label-free quantification (LFQ) algorithm and advanced annotation tools to find differences between the studied groups. In both analyses, we found the number of proteins previously mentioned as potential DCM biomarkers and proteins and biological terms frequently discussed as being connected to the pathogenesis of the disease.

## Experimental Procedures

### Study Population

The prospective study included 49 patients with recent onset DCM from three cardiac centers in the Czech Republic: University Hospital Hradec Králové, Institute for Clinical and Experimental Medicine Prague, and University Hospital Olomouc. The samples were collected during 2019–2021. DCM was diagnosed according to the recommendations of the European Society of Cardiology^2,3^ (ESC). DCM was defined as the presence of left ventricle dilatation and global or regional systolic dysfunction unexplained solely by abnormal loading conditions (e.g., hypertension, valve disease, and congenital heart disease) or coronary artery disease. All patients underwent systematic evaluation, which included: clinical evaluation, pedigree analysis, electrocardiography and Holter monitoring, laboratory tests, and cardiac magnetic resonance imaging. Genetic testing was not performed. Inclusion criteria were as follows: 1) recent onset nonischemic DCM defined according to latest guidelines, 2) LVEF ≤ 40% by echocardiography, and 3) symptoms ≤2 months in duration. Exclusion criteria covered secondary forms of DCM (tachycardiomyopathy, substance abuse, cardiotoxic agents, systemic autoimmune disease, peripartum cardiomyopathy, endocrine diseases, active myocarditis, and DCM secondary to hypertension). In accordance with the guidelines of ESC,^2,3^ the patients fulfilling diagnostic criteria for clinically suspected myocarditis as well as the patients with another disease or condition that could affect the results of biochemical and proteomic parameters (chronic liver and kidney disease, immunopathology, cancer, etc.) were not enrolled into the study.

### Study Protocol

At baseline, all subjects underwent clinical examination, electrocardiogram, echocardiography, coronary arteriography, and cardiac magnetic resonance, and peripheral venous blood samples were taken. Management of the patients included pharmacological treatment as well as nonpharmacological procedures—implantable cardioverter-defibrillator (ICD) or cardiac resynchronization therapy (CRT) — according to the ESC guidelines.^15,16^ Clinical evaluation, electrocardiography, and echocardiography were then performed 12 months after the first examination. During the 12-months period, the patients were further examined in 3-months intervals with the aim to maximize optimal medical treatment (uptitration, indication of ICD or CRT-P/D). Echocardiography was performed by experienced operators in accordance with European Association of Cardiovascular Imaging^17^ and American Society of Echocardiography guidelines.^18^ LVEF was assessed using Simpson’s biplane method. The control group consisted of 25 healthy persons (with the age and sex distribution matching the DCM patients) who were examined at the first Department of Internal Medicine — Cardioangiology, University Hospital Hradec Králové with the aim of excluding cardiovascular diseases (personal history, physical examination, electrocardiogram, and echocardiography) and other diseases that could affect the monitored parameters.

Baseline LVEF (LVEF0), LVEF after one year of treatment (LVEF1), and their difference (LVEF1 – LVEF0 = Δ*L*VEF) were considered to assort the second analytical set to further compare the DCM patients according to their treatment response. Only the patients with LVEF0 ≤ 20% were here enrolled. Poor response without left ventricle reverse remodeling (LVRR−) was defined as Δ*L*VEF < 10% and alsoincluded the patients where LVEF1 measurement could not be taken because the patient was indicated with mechanical circulatory support (MCS), underwent orthotopic heart transplantation, or died. In those cases, LVEF1 was replaced by 0 for the purpose of Δ*L*VEF calculation. Rest of the subgroup with Δ*L*VEF > 10% was considered as improved after the treatment (LVRR+).

The clinical and demographic characteristics of the studied groups were compared by a chi-square test of homogeneity (for the categorical variables) or by an unpaired two-sample Student’s *t* test (for the continuous variables). The *p*-value lower than 0.05 was considered significant.

### Ethic Statement

The study was approved by the ethical committee of the University Hospital Hradec Králové. The trial was conducted following the principles of the Declaration of Helsinki.

### Plasma Sample Collection

After 12 h of fasting, two blood samples of peripheral blood (e.g., from cubital vein) were withdrawn (one for proteomic analysis and one for biochemical analysis). Samples for proteomic analysis were collected using the BD P100 blood collection tubes (Becton Dickinson) containing K_2_EDTA anticoagulant, proprietary protease inhibitor cocktail and equipped with a mechanical separator. Within 1 h from sampling, the collection tubes were centrifuged (2500 × g, 20 min, 22 °C, ankle rotor), and separated plasma was transferred to a new tube. Residual cells were then briefly pelleted (same conditions, 5 min), and cleared plasma was aliquoted and stored at −80 °C.

### Abundant Protein Depletion

Depletions were performed using High Select Top14 Abundant Protein Depletion Mini Spin Columns (Thermo Scientific). The columns were equilibrated at RT before use. Ten μL of plasma were added to the resin suspension, and the columns were incubated with gentle end-overend mixing for 1 h at RT. The flowthrough was collected in new vials by centrifugation (1000 × g, 2 min, RT) and stored at −80 °C. Protein concentrations in the depleted samples were measured by a modified BCA assay (Sigma–Aldrich), and the effectivity of the depletion was further evaluated by SDS-PAGE of the individual plasma samples.

### MS Sample Preparation

Depleted plasma samples were diluted with 10 mM phosphate buffered saline to 0.167 μg/μL, and proteins were solubilized by boiling for 10 min with 1% (w/v) sodium dodecyl sulfate. Proteins were then reduced with 200 mM *tris*(2-carboxyethyl)phosphine at 60 °C for 1 h, alkylated with 375 mM iodoacetamide at RT for 30 min in darkness, and precipitated by six volumes of ice-cold acetone at −20 °C overnight. Proteins were pelleted (8000 × g, 10 min, 4 °C) and dried on air. Protein pellets were solubilized in 100 mM TEAB with 1% (w/v) sodium deoxycholate (SDC) and digested with sequencing grade trypsin (Promega) at 37 °C overnight. SDC was removed following the modified phase transfer protocol:^19^ the samples were acidified with trifluoroacetic acid (TFA) to pH < 3, pelleted (5000 × g, 8 min, 4 °C), and the pellet was rinsed with 1% TFA (same conditions). Combined supernatants were extracted 5× with ethyl-acetate by vigorous vortexing and brief spin. Residual ethyl-acetate was removed by short vacuum drying. The peptides were desalted using Empore C18-SD (4 mm/1 mL) extraction cartridges (Sigma-Aldrich), dried and stored at −40 °C until the MS analysis.

### Liquid Chromatography and Mass Spectrometry Analysis

Liquid chromatography and tandem mass spectrometry analysis (LC-MS/MS) was performed on the Ultimate 3000 RSLCnano System (Dionex) coupled online through Nanospray Flex ion source with a Q Exactive mass spectrometer (Thermo Scientific). Peptide mixtures dissolved in 2% acetonitrile (ACN)/0.05% TFA were loaded onto a capillary trap column (C18 PepMap100, 3 μm, 100, 0.075 × 20 mm^2^) and separated on the capillary column (C18 PepMap RSLC, 2 μm, 100, 0.075 × 150 mm^2^; both Dionex) by step linear gradient of mobile phase B (80% ACN/0.1% formic acid) over mobile phase A (0.1% formic acid) from 4% to 34% B during 68 min and from 34% to 55% B during 21 min at a flow rate of 300 nL/min. The column was kept at 40 °C, and the eluent was monitored at 215 nm. The mass spectrometer operated in the positive ion mode performing survey MS (at 350–1650 *m*/*z*) and data-dependent MS/MS scans of 12 most intense precursors with a dynamic exclusion window of 30 s and an isolation window of 1.6 Da. MS scans were acquired with a resolution of 70 000 from 10^6^ accumulated charges, and maximum fill time was 100 ms. Normalized collision energy for higher energy collisional dissociation fragmentation was 27 units. MS/MS spectra were acquired with a resolution of 17 500 from 10^5^ accumulated charges, and the maximum fill time was 100 ms. Each sample was measured once, and to minimize batch effect, the order of samples was randomized.

### Protein Identification and Label-Free Quantification

Raw files were searched in MaxQuant v2.4.2.0^20^ against the human reference proteome set downloaded from UniProt in May 2023 (20,603 sequences). MaxQuant implemented database of common contaminants was enabled after deletion of the bovine serum proteins. Peptide modifications were set as follows: fixed modification: carbamidomethylation of cysteine; variable modifications: oxidation of methionine, acetylation of protein N-term, and Gln to pyro-Glu; maximum number of variable modifications was 5 per peptide. Label-free quantification was enabled by treating the individual samples as separate experiments. Matching between runs was on with a match time of 1 min and an alignment time window of 20 min. Other MaxQuant parameters were set as default.

The mass spectrometry proteomics data have been deposited to the ProteomeXchange Consortium via the PRIDE^21^ partner repository with the data set identifier PXD046288 and 10.6019/PXD046288.

### Proteomic Data Analysis

Downstream analysis was performed in Perseus software v2.0.10.0^22^ on the data imported from the MaxQuant “proteinGroups.txt” output tables. The LFQ intensity values were imported, data were filtered from potential contaminants, reverse hits, and proteins only identified by site, log2 transformed, and grouped according to sample classification. Proteins with less than 50% valid quantification values and less than 2 peptides were filtered off, and missing values were imputed (width 0.3, down shift 1.8, separately for each column). Relative protein quantity differences between sample groups and their statistical significances were calculated by two-sample Student’s *t* tests with truncations resulting from Benjamini-Hochberg FDR (FDR = 0.05, S0 = 0.1). In the case of the LVRR– vs LVRR+ comparison, a less stringent truncation based on *p*-value (*p* = 0.05, S0 = 0.1) was used for the purpose of PCA analysis and the functional enrichment analysis.

### Protein Annotation and Functional Enrichment Analysis

Protein annotation and identification of enriched biological themes was performed using the database for annotation, visualization, and integrated discovery (DAVID).^23,24^ The lists of significantly up-regulated and down-regulated proteins from each comparison were searched separately against the background of all identified proteins (before *t* test filtration). The presented enriched themes were limited to the gene ontology (GO) biological process terms with *p*-value ≤ 0.05. Cytoscape software v3.10.0^25^ together with the StringApp application^26^ were used to visualize the potential interaction networks among the proteins from the enriched terms.

### Logistic Regression with Elastic Net Optimization

A predictive statistical approach of logistic regression with elastic net optimization was applied for the proteins obtained by the evaluation of LC-MS/MS data in MaxQuant and Perseus software. The lists of significantly deregulated proteins with their intensity information were processed using statistical package “glmnet” (version 4.1–8) in the RStudio program (v. 2023.09.1 + 494) running R programming language (v. 4.3.2). Briefly, the samples in both data sets were randomly divided into training (75%) and testing (25%) groups. The training group data were used for predictive model computation (parameter alpha set for 1) with an optimized lambda coefficient. The computed model prediction was then used for testing group samples, and the results were confronted with the experimental sample information. Random selection of the training sample group with the corresponding model computation was repeated 100 times in order to estimate an average model prediction efficiency. The R language commands for the logistic regression data processing are described in the Supplementary R-script of this manuscript.

## Results

### Patient Group Characterization

The patients’ characteristics are listed in Table 1. The discovery set for the first proteomic analysis comprised 49 patients with recent onset DCM. The average LVEF at admission (LVEF0) of the whole DCM group was 20.3% ± 6.5, and the group enrolled all the patients regardless of their treatment response or heart damage progress. This group was compared to the healthy control blood donors (*n* = 25) without any history of cardiovascular disease. The analysis set 2 was assorted from the first set to subsume only the patients with the worst initial clinical state (LVEF0 values ≤ 20%). It comprised 34 patients, which were further subdivided according to the disease development. The LVRR– were the patients without significant LVRR (Δ*L*VEF values < 10%, *n* = 19), and they involved also those who underwent heart transplantation, were indicated to MCS, or deceased within the first year. The patients with positive treatment reaction (LVRR+, *n* = 15) involved those whose LVEF increased at least 10% after the first year, and the average Δ*L*VEF of this subgroup was 34.1% ± 9.1.

**Table 1:** Characteristics of the Study Population^a^,^b^

	analysis set 1		analysis set 2	
patient characteristics	control (*n* = 25)	DCM (*n* = 49)	LVRR+ (*n* = 15)	LVRR– (*n* = 19)
age (±SD)	59.5 ± 2.9	51.3 ± 14.7^c^	52.3 ± 11.7	44.8 ± 13.6^d^
males (*n*, %)	21 (84.0)	35 (71.4)^d^	11 (73.3)	15 (78.9)^d^
smoking (*n*, %)		11 (22.4)	5 (33.3)	9 (47.4)^d^
diabetes mellitus (*n*, %)		12 (24.5)	4 (26.7)	4 (21.1)^d^
arterial hypertension (*n*, %)		19 (38.8)	7 (46.7)	3 (15.8)^e^
hyperlipidemia (*n*, %)		23 (46.9)	8 (53.3)	9 (47.4)^d^
atrial fibrillation (*n*, %)		11 (22.4)	6 (40.0)	0^e^
admission data:				
heart rate (bpm, ± SD)		96.1 ± 23.2	96.3 ± 20.1	102.6 ± 15.5^d^
blood pressure (mmHg, ± SD)		112.9 ± 26.3/80.6 ± 18.4	116,6 ± 15.1/83.1 ± 13.7	110.1 ± 35.4^d^/73.1 ± 22.5^d^
LVEF0 (±SD)		20.3 ± 6.5	15.9 ± 3.8	18.0 ± 3.2^d^
creatine (μmol/L, ± SD)		99.8 ± 29.7	97.7 ± 29.3	107.9 ± 35.4^d^
GFR (CKD-EPI, mL/s, ± SD)		1.2 ± 0.4	1.2 ± 0.4	1.3 ± 0.3^d^
NYHA class (*n*, %)				
II		23 (46.9)	8 (53.3)	8 (42.1)^d^
III		17 (34.7)	5 (33.3)	7 (36.8)^d^
IV		9 (18.4)	2 (13.3)	4 (21.1)^d^
cardiogenic shock (*n*, %)		2 (4.1)	0	2 (10.5)^d^
discharge therapy (*n*, %):				
beta-blockers		44 (89.7)	12 (80.0)	17 (89.5)^d^
ACEi		28 (57.1)	9 (60.0)	11 (57.9)^d^
ARNi		12 (24.3)	2 (13.3)	8 (42.1)^d^
MRA		35 (71.4)	11 (73.3)	15 (78.9)^d^
diuretics		34 (69.4)	11 (73.3)	14 (73.7)^d^
ICD		23 (46.9)	3 (20.0)	8 (42.1)^d^
CRT-P		12 (24.5)	1 (6.7)	8 (42.1)^e^
12 months after admission:				
heart transplantation (*n*, %)		4 (8.2)	0	4 (21.1)^d^
MCS (*n*, %)		3 (6.1)	0	3 (15.8)^d^
deceased (*n*, %)		3 (6.1)	0	3 (15.8)^d^
LVEF1 (±SD)		33.8 ± 22.5	50.0 ± 9.3	10.3 ± 11.0^f^
ΔLVEF (±SD)		13.5 ± 21.3	34.1 ± 9.1	–7.7 ± 10.7^f^

a The statistical significance (*p* < 0.05) was calculated by chi-square test of homogeneity (for the categorical variables), or by unpaired two-sample Student’s T-test (for the continuous variables).

b SD: standard deviation; LVEF0: left ventricular ejection fraction at admission; LVEF1: LVEF after one year; ΔLVEF = LVEF1 – LVEF0; GFR: glomerular filtration rate; ACEi: angiotensin converting enzyme inhibitor; ARNi: angiotensin receptor-neprilysin inhibitor; MRA: mineralocorticoid receptor antagonist; ICD: implantable cardioverter-defibrillator; CRT-P: cardiac resynchronization therapy; MCS: mechanical circulatory support.

c significantly different from healthy control (*p* < 0.05).

d ns: not significant.

e Significantly different from LVRR+ (*p* < 0.05).

f The LVRR– group contains patients where the LVEF1 measurement could not be taken due to reasons described in the text; in these cases, LVEF1 = 0 and statistical significance could not be calculated.

### Proteomic Comparison of DCM vs Healthy Control

**Figure 1:**
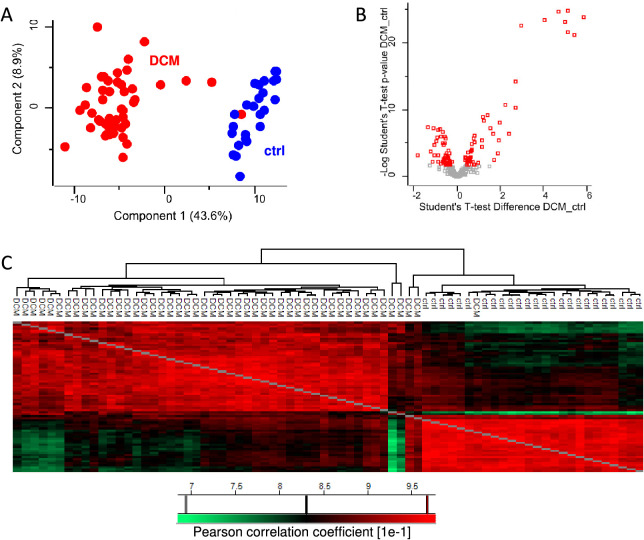
Proteomic comparison of healthy control vs DCM plasma samples. (A) Principal component analysis separated clearly the controls from the DCM samples. (B) Volcano plot of all identified proteins, significantly deregulated proteins are highlighted in red. Student’s *t* test difference (Dcm_ctrl) corresponds to the log2 fold change of DCM over ctrl. (C) Hierarchical clustering of the samples based on their reciprocal Pearson correlation coefficients.

The first proteomic comparison was focused on searching for differences between healthy control plasma samples and those from patients with DCM. In this analysis, the DCM group included all the DCM patients (*n* = 49) regardless of the disease progress. The proteomic analysis based on the LFQ approach identified 288 proteins with sufficient quantification characteristics. In total, 97 proteins were found to be significantly differently expressed between the control and DCM groups; of them, 45 proteins were up-regulated and 52 were down-regulated in the DCM samples. Figure 1B shows the volcano plot of identified proteins with significantly regulated proteins. The patient versus control groups were clearly distinct which is depicted on the principal component analysis (PCA) plot (Figure 1A) and the heat map of their hierarchical clustering according to the reciprocal correlation coefficients (Figure 1C). The list of significantly deregulated proteins is presented in Table 2, and the full list including the data from the proteomic analysis is in Supplementary Table 1. A logistic regression with elastic net optimization for random selection of training sample group with the significantly deregulated proteins provided 96% (min. 83%, max. 100%) average agreement of the model with the experimental data (see Supplementary Figure 1). Corresponding ROC plots further illustrating the prediction power of computed models are shown in Supplementary Figure 2 (top panel).

**Table 2:** List of Proteins Significantly Deregulated between the Control and DCM Group^a^

protein ID	gene name	protein name	Student’s *t* test difference	–log p
P36980	CFHR2	complement factor H-related protein 2	5.84	23.92
P01860	IGHG3	Ig gamma-3 chain C region	5.10	21.67
P01344	IGF2	insulin-like growth factor II	5.09	24.92
P27918	CFP	properdin	4.97	23.16
P54108	CRISP3	cysteine-rich secretory protein 3	4.04	23.47
P02760	AMBP	protein AMBP	2.94	22.68
Q12805	EFEMP1	EGF-containing fibulin-like extracellular matrix protein 1	2.70	10.34
P59666	DEFA3	neutrophil defensin 3	2.67	14.31
P02741	CRP	C-reactive protein	2.40	6.43
Q86VB7	CD163	Scavenger receptor cysteine-rich type 1 protein M130	2.20	10.85
P18065	IGFBP2	insulin-like growth factor-binding protein 2	1.98	8.14
P02776	PF4	platelet factor 4	1.93	5.50
P0DJI8	SAA1	serum amyloid A-1 protein	1.87	3.50
P23229	ITGA6	integrin alpha-6	1.69	2.54
P98160	HSPG2	basement membrane-specific heparan sulfate proteoglycan core protein	1.64	6.46
P08637	FCGR3A	low affinity immunoglobulin gamma Fc region receptor III-A	1.53	7.28
P20851	C4BPB	C4b-binding protein beta chain	1.41	9.29
P00747	PLG	plasminogen	1.21	8.34
P00734	F2	prothrombin	1.18	9.00
Q16853	AOC3	membrane primary amine oxidase	1.13	5.50
P24821	TNC	tenascin	1.13	4.48
P05109	S100A8	protein S100-A8	0.97	2.06
P04406	GAPDH	glyceraldehyde-3-phosphate dehydrogenase	0.81	1.77
P01009	SERPINA1	alpha-1-antitrypsin	0.81	5.25
P02745	C1QA	complement C1q subcomponent subunit A	0.79	6.18
P02750	LRG1	leucine-rich alpha-2-glycoprotein	0.77	5.82
O43866	CD5L	CD5 antigen-like	0.75	1.86
Q15848	ADIPOQ	adiponectin	0.71	2.21
Q9Y6R7	FCGBP	IgGFc-binding protein	0.67	3.95
P39060	COL18A1	collagen alpha-1(XVIII) chain	0.67	4.05
P05156	CFI	complement factor I	0.64	4.89
Q06033	ITIH3	interalpha-trypsin inhibitor heavy chain H3	0.62	3.78
P02675	FGB	fibrinogen beta chain	0.62	2.28
P02763	ORM1	alpha-1-acid glycoprotein 1	0.60	2.91
P02671	FGA	fibrinogen alpha chain	0.58	2.84
P00738	HP	haptoglobin	0.57	2.67
P02749	APOH	beta-2-glycoprotein 1	0.53	2.29
P13598	ICAM2	intercellular adhesion molecule 2	0.52	1.87
P04003	C4BPA	C4b-binding protein alpha chain	0.51	3.59
P61626	LYZ	lysozyme C	0.46	2.41
P19320	VCAM1	vascular cell adhesion protein 1	0.44	1.90
P05362	ICAM1	intercellular adhesion molecule 1	0.43	2.08
P01011	SERPINA3	alpha-1-antichymotrypsin	0.41	2.86
P00450	CP	ceruloplasmin	0.37	2.54
P08603	CFH	complement factor H	0.36	2.94
O75882	ATRN	attractin	–0.26	2.30
P22105	TNXB	tenascin-X	–0.33	1.92
P04180	LCAT	phosphatidylcholine-sterol acyltransferase	–0.34	2.15
P22352	GPX3	glutathione peroxidase 3	–0.35	1.82
Q16610	ECM1	extracellular matrix protein 1	–0.36	2.03
P02743	APCS	serum amyloid P-component	–0.36	1.95
P07225	PROS1	vitamin K-dependent protein S	–0.37	2.28
Q12913	PTPRJ	receptor-type tyrosine-protein phosphatase eta	–0.37	2.39
P03952	KLKB1	plasma kallikrein	–0.38	3.51
Q9UNW1	MINPP1	multiple inositol polyphosphate phosphatase 1	–0.40	1.84
Q01459	CTBS	di-N-acetylchitobiase	–0.40	2.06
P43251	BTD	biotinidase	–0.42	3.92
P00742	F10	coagulation factor X	–0.43	2.87
P36955	SERPINF1	pigment epithelium-derived factor	–0.43	3.95
P51884	LUM	lumican	–0.44	3.07
P05160	F13B	coagulation factor XIII B chain	–0.45	2.89
P04070	PROC	vitamin K-dependent protein C	–0.47	2.48
P02654	APOC1	apolipoprotein C–I	–0.50	2.58
O00533	CHL1	neural cell adhesion molecule L1-like protein	–0.51	1.80
Q9UK55	SERPINA10	protein Z-dependent protease inhibitor	–0.51	3.47
P04196	HRG	histidine-rich glycoprotein	–0.51	2.86
P29622	SERPINA4	kallistatin	–0.52	3.18
Q92820	GGH	gamma-glutamyl hydrolase	–0.52	1.85
P49747	COMP	cartilage oligomeric matrix protein	–0.54	1.92
P51659	HSD17B4	peroxisomal multifunctional enzyme type 2	–0.55	2.12
P49908	SEPP1	selenoprotein P	–0.56	5.75
P12259	F5	coagulation factor V	–0.57	5.02
P12830	CDH1	cadherin-1	–0.57	2.78
Q92954	PRG4	proteoglycan 4	–0.58	2.06
P02647	APOA1	apolipoprotein A-I	–0.61	6.02
P06276	BCHE	cholinesterase	–0.61	4.82
P27169	PON1	serum paraoxonase/arylesterase 1	–0.64	2.57
Q15166	PON3	serum paraoxonase/lactonase 3	–0.64	2.72
Q9UNN8	PROCR	endothelial protein C receptor	–0.66	2.21
P27487	DPP4	dipeptidyl peptidase 4	–0.66	2.32
P80108	GPLD1	phosphatidylinositol-glycan-specific phospholipase D	–0.66	5.07
P02652	APOA2	apolipoprotein A-II	–0.69	6.68
P02751	FN1	fibronectin	–0.75	1.96
P05154	SERPINA5	plasma serine protease inhibitor	–0.77	6.00
Q9NZJ4	SACS	sacsin	–0.82	7.18
Q9HDC9	APMAP	adipocyte plasma membrane-associated protein	–0.89	2.26
P00488	F13A1	coagulation factor XIII A chain	–0.89	6.98
Q6UXB8	PI16	peptidase inhibitor 16	–0.90	3.23
O75636	FCN3	ficolin-3	–0.93	5.96
P06396	GSN	gelsolin	–0.95	5.27
Q96KN2	CNDP1	beta-Ala-His dipeptidase	–1.04	4.76
P22891	PROZ	vitamin K-dependent protein Z	–1.07	3.26
P06727	APOA4	apolipoprotein A-IV	–1.11	7.25
P08709	F7	coagulation factor VII	–1.15	2.25
Q9BUN1	MENT	protein MENT	–1.23	3.77
Q9NQ79	CRTAC1	cartilage acidic protein 1	–1.38	7.43
Q8WZ42	TTN	titin	–1.84	3.25

a Student’s T-test difference corresponds to the log2 fold change of DCM over ctrl.

### Proteomic Comparison of DCM Patients According to Response to Treatment

**Figure 2:**
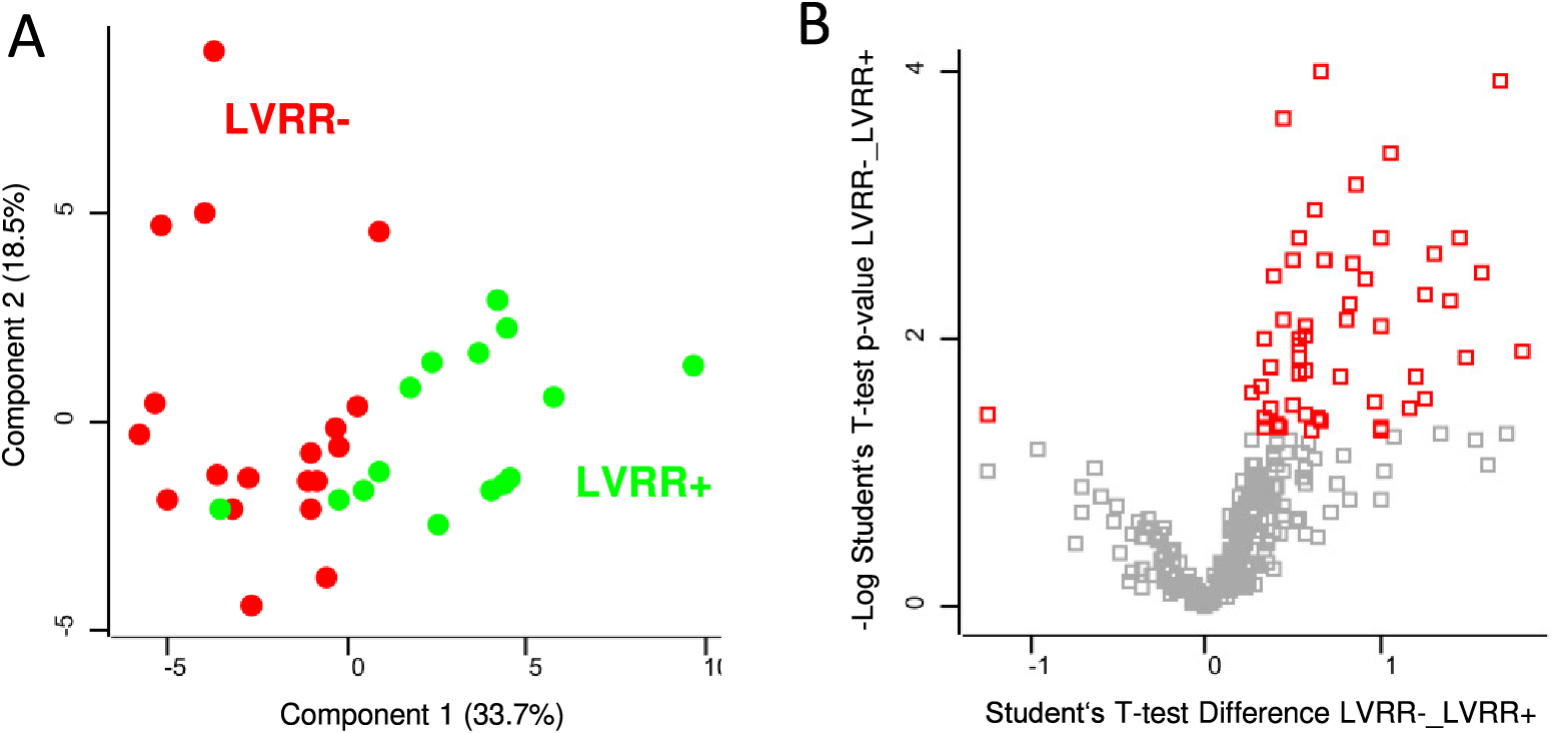
Proteomic comparison of the LVRR– vs LVRR+ DCM plasma samples. (A) Principal component analysis. (B) Volcano plot of all identified proteins, significantly deregulated proteins are highlighted in red. Student’s *t* test difference (LVRR–_LVRR+) corresponds to the log2 fold change of LVRR– over LVRR+.

The second proteomic comparison was focused on the DCM patient group. The whole DCM group was rather homogeneous and did not reveal any differently expressed proteins. For this reason, only the patients with the most severe initial state (LVEF0 ≤ 20%) were selected for this part of the study. The nonresponding LVRR– subgroup combined patients who reacted to the treatment with no or only minimal LVEF improvement (Δ*L*VEF < 10%), those with mechanical support, heart transplantation, and those who died during the first year. Rest of the subgroup with Δ*L*VEF ≥ 10% were considered as responders (LVRR+). The described subgrouping allowed for finding differently expressed proteins, as depicted on the PCA and volcano plot (Figure 2). The distinction was not as clear as in the comparison of DCM patients with healthy control, but it was satisfactory considering that the differences between individual patients are expected to be lower and may be further distorted by diverse etiologies of DCM and other aspects of the patients’ health condition.

The LFQ analysis revealed 299 proteins, 45 of them were significantly differently expressed (*p* value ≤0.05) between LVRR– and LVRR+ subgroups, 44 of them being up-regulated, and only 1 down-regulated in the LVRR– samples. The list of significantly different proteins is presented in Table 3, and the full list including the data from the proteomic analysis is in Supplementary Table 2. A logistic regression with elastic net optimization for random selection of training sample group with the significantly deregulated proteins (based on 0.05 FDR selection, proteins highlighted in Table 3) provided 88% (min. 57%, max. 100%) average agreement of the model with the experimental data (see Supplementary Figure 1). Corresponding ROC plots further illustrating the prediction power of computed models are shown in Supplementary Figure 2 (bottom panel).

**Table 3:** List of Proteins Significantly Deregulated between LVRR– and LVRR+ DCM Subgroups (*p* value ≤ 0.05)^a^

protein ID	gene name	protein name	student’s *t* test difference	–log p
P05062	ALDOB	fructose-bisphosphate aldolase B	1.80	1.90
**P01860**	**IGHG3**	**Ig gamma-3 chain C region**	**1.57**	**2.49**
P24298	GPT	Alanine aminotransferase 1	1.49	1.85
**P01591**	**IGJ**	**immunoglobulin J chain**	**1.45**	**2.74**
**P01871**	**IGHM**	**Ig mu chain C region**	**1.31**	**2.63**
**P01780**	**IGHV3–7**	**immunoglobulin heavy variable**3–7	**1.25**	**2.32**
**P06310**	**IGKV2–30**	**immunoglobulin kappa variable**2–30	**1.06**	**3.40**
P02776	PF4	platelet factor 4	1.00	1.30
**P0DP04**	**IGHV3–43D**	**immunoglobulin heavy variable**3–43**D**	**1.00**	**2.76**
P01857	IGHG1	Ig gamma-1 chain C region	1.00	2.08
P01859	IGHG2	Ig gamma-2 chain C region	0.96	1.54
P0DOY3	IGLC3	immunoglobulin lambda constant 3	0.91	2.44
**P02786**	**TFRC**	**transferrin receptor protein 1**	**0.86**	**3.15**
P01834	IGKC	Ig kappa chain C region	0.84	2.56
P01876	IGHA1	Ig alpha-1 chain C region	0.82	2.27
O00533	CHL1	neural cell adhesion molecule L1-like protein	0.81	2.14
Q9NPH3	IL1RAP	interleukin-1 receptor accessory protein	0.77	1.72
P07359	GP1BA	platelet glycoprotein Ib alpha chain	0.67	2.58
**P14151**	**SELL**	**L-selectin**	**0.67**	**3.99**
P23470	PTPRG	receptor-type tyrosine-protein phosphatase gamma	0.66	1.38
P27918	CFP	properdin	0.64	1.41
P01023	A2M	alpha-2-macroglobulin	0.61	2.97
Q15485	FCN2	ficolin-2	0.60	1.31
P54108	CRISP3	cysteine-rich secretory protein 3	0.57	2.02
P43121	MCAM	cell surface glycoprotein MUC18	0.56	2.10
Q16853	AOC3	membrane primary amine oxidase	0.54	1.94
Q01459	CTBS	di-N-acetylchitobiase	0.54	1.73
P02787	TF	serotransferrin	0.53	2.00
P22352	GPX3	glutathione peroxidase 3	0.53	1.85
P00450	CP	ceruloplasmin	0.53	2.74
Q12913	PTPRJ	receptor-type tyrosine-protein phosphatase eta	0.50	2.60
P02760	AMBP	protein AMBP	0.49	1.50
Q14520	HABP2	hyaluronan-binding protein 2	0.44	2.14
P03951	F11	coagulation factor XI	0.44	3.64
Q9Y5Y7	LYVE1	lymphatic vessel endothelial hyaluronic acid receptor 1	0.43	1.34
P08637	FCGR3A	low affinity immunoglobulin gamma Fc region receptor III-A	0.40	1.35
P03952	KLKB1	plasma kallikrein	0.39	2.48
P25311	AZGP1	zinc-alpha-2-glycoprotein	0.37	1.47
P81605	DCD	dermcidin	0.37	1.78
P02774	GC	vitamin D-binding protein	0.34	2.00
P22105	TNXB	tenascin-X	0.33	1.34
P49908	SEPP1	selenoprotein P	0.33	1.41
P43251	BTD	biotinidase	0.31	1.64
O75882	ATRN	attractin	0.26	1.59
P18428	LBP	lipopolysaccharide-binding protein	–1.24	1.44

a Student’s T-test difference corresponds to the log2 fold change of LVRR– over LVRR+ DCM patients. Proteins highlighted in bold satisfied the criteria of FDR ≤ 0.05 and were further used for logistic regression with elastic net optimization.

### Functional Annotation Enrichment and Protein–Protein Interactions of the Regulated Proteins

**Figure 3:**
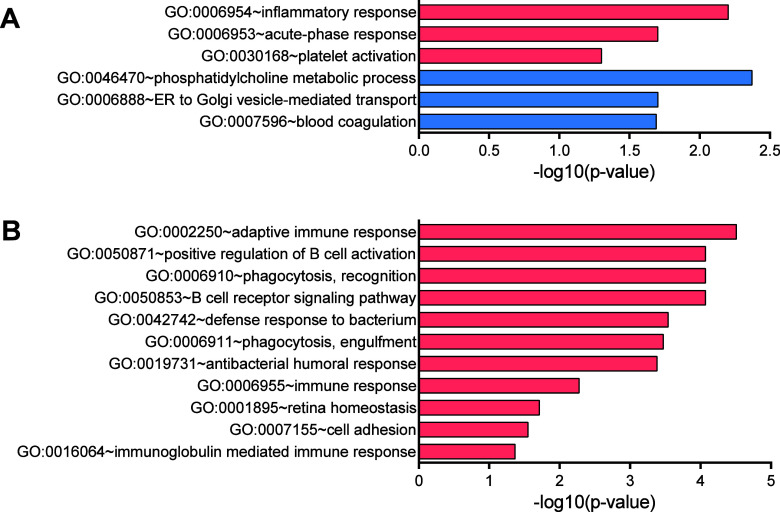
Functional enrichment of GO “biological process” terms. (A) Healthy control vs DCM, red: up-regulated, blue: down-regulated in DCM patients. (B) LVRR– vs LVRR+, red: up-regulated in LVRR–.

**Figure 4:**
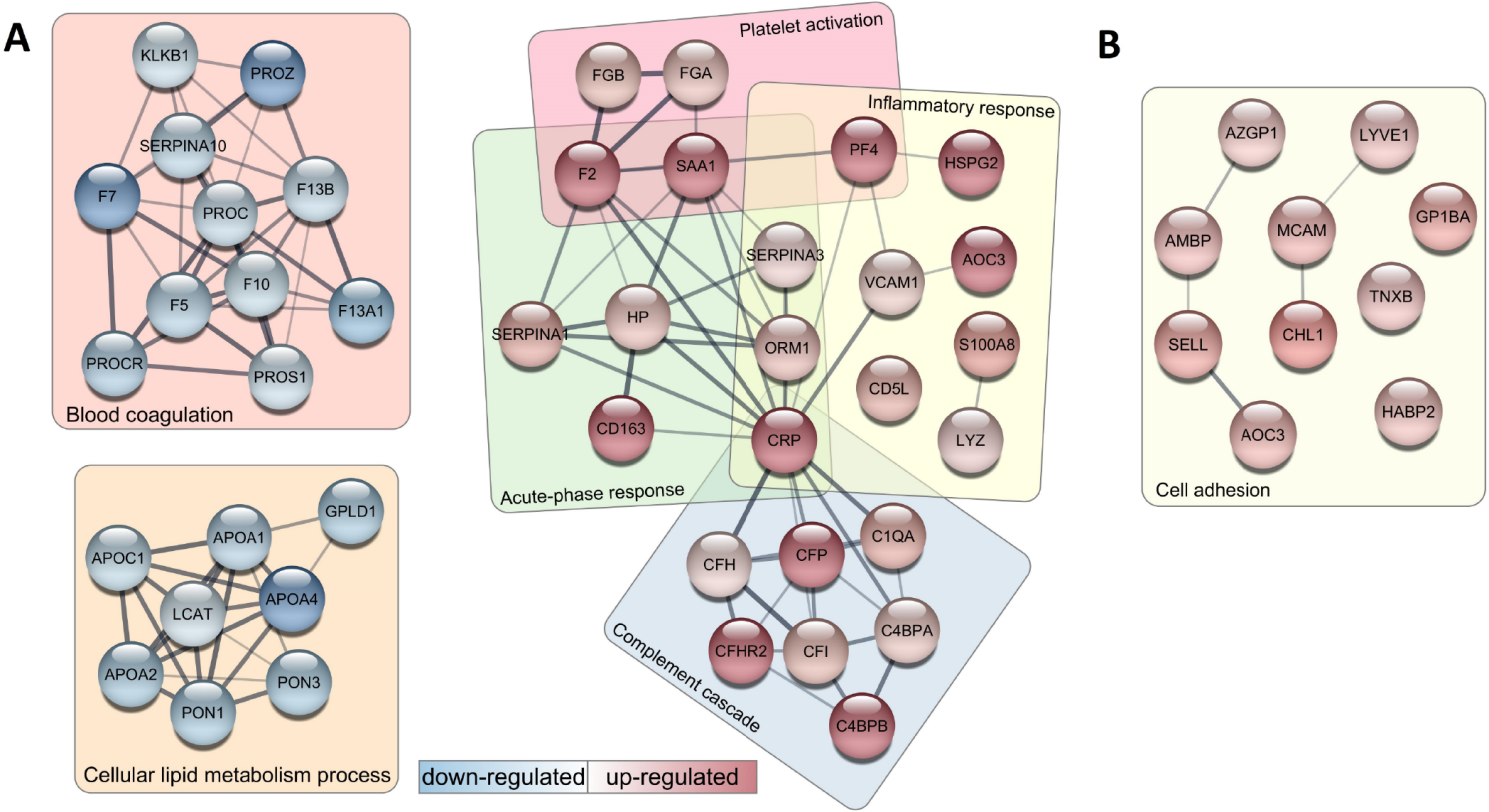
Selected regulated proteins from the enriched biological terms and their potential interaction networks visualized by StringApp in Cytoscape software. (A) Healthy control vs DCM, red: up-regulated, blue: down-regulated in DCM patients. (B) LVRR- vs LVRR+, red: up-regulated in LVRR-. Only the biological process of cell adhesion is here pictured because other GO terms involve immunoglobulins that are not annotated in String.

We further performed functional enrichment analysis to find relevant regulated biological themes. The annotation of differently expressed proteins was limited to their GO biological process categories (see Figure 3). The subgroups of up- and down-regulated proteins were analyzed separately against the individual backgrounds of the identified proteins to avoid false detection of huge amounts of incidentally enriched terms. In the LVRR– vs LVRR+ DCM comparison, only up-regulated terms were searched because there was only one protein found to be down-regulated. The full list of enriched annotation terms is in Supplementary Table 3. The potential protein interaction networks among the regulated proteins and terms are shown in Figure 4.

The proteins up-regulated in DCM over control were significantly enriched in terms collectively involved in the immune response and wound healing (the inflammatory and acute-phase response, platelet activation, and complement cascade). On the contrary, the down-regulated proteins in DCM were enriched in blood coagulation and lipid metabolism. The proteins up-regulated in the nonresponding LVRR– DCM subgroup were highly enriched in immunoglobulins and immunoglobulin-related GO terms. Besides the immunoglobulins, the LVRR– up-regulated proteins were also enriched in the biological process of cell adhesion.

## Discussion

In the present study, we performed a comprehensive quantitative proteomic analysis of DCM patients’ plasma samples. In the first part of the study, we searched for the differences against the healthy control group and found several proteins and biological processes to be significantly regulated. Some of the proteins have been considered as potential DCM biomarkers previously, although most of the dysregulated proteins in DCM patients indicated a nonspecific response corresponding to any serious cardiovascular disease. Nevertheless, DCM is a progressive cardiac condition. Even with discharge therapy, it is a relevant cause of heart failure, and the prognosis of individual patients is problematic. For this reason, there is still a need for new biomarkers that would not only serve for DCM diagnosis but also have better predictive potential. The currently known DCM protein biomarkers do not provide sufficient power to estimate DCM progression. Therefore, we further observed the clinical fate of the DCM patients’ one year after admission. We divided them according to their treatment response into subgroups of responders and nonresponders and searched for proteins that would help to predict the patient’s fate or to elucidate the molecular consequences of the disease progression.

The DCM samples compared to the healthy control revealed a number of deregulated proteins with high discriminative accuracy to distinguish DCM cases from controls. The up-regulated proteins were mostly involved in the inflammatory response and wound healing, including the complement cascade system. Namely, the complement proteins belonged to the topmost up-regulated proteins in DCM patients together with the immunoglobulin gamma-3 chain C region (IGHG3). The deposition of IgG and components of complement on cardiomyocytes has been described previously as a cause of damage of the cardiac tissue contributing to the pathogenesis of DCM.^27,28^ Similarly, the autoimmunity and autoantibodies have been widely discussed as possible pathogenesis mechanism of idiopathic DCM.^29^ The inflammatory response proteins, C-reactive protein (CRP) and scavenger receptor cysteine-rich type 1 protein M130 (CD163), are connected to inflammatory cells infiltration in the heart tissue and were found to be elevated in myocardial biopsies of DCM patients in several studies.^30,31^ Taken together, our results confirm the important role of immuno-inflammatory cascades in development of DCM.^32^

Some of the proteins up-regulated in DCM patients in our study were previously studied as potential DCM markers. For example, tenascin (TNC) was found ca. 2x up-regulated in DCM patients over control. TNC was proposed as an effective biomarker of DCM,^33^ moreover, TNC level in DCM patients’ plasma correlated with successful treatment and treatment-related reverse ventricular remodeling.^34,35^

Other proteins up-regulated in DCM over control are known to be involved more generally in cardiovascular disorders or present some risk factors leading to cardiovascular diseases. One of them is the protein AMBP (a precursor of alpha-1-microglobulin and bikunin).^36^ In our study, it was highly up-regulated in DCM over control, and it was also slightly higher in LVRR- than in the LVRR+. Similarly, the membrane primary amine oxidase (AOC3) and platelet factor 4 (PF4) were up-regulated in DCM patients compared to control and in patients with poor response over those who improved, as well. Hence, the upregulation of AMPB, AOC3 and PF4 can also indicate worse prognosis in DC patients concerning the response to treatment. Besides PF4, we have also observed elevated levels of other proteins involved in platelet activation such as serum amyloid A-1 protein (SAA1), prothrombin (F2), and fibrinogen alpha and beta chain (FGA, FGB). Platelet activation and thrombin activation together with fibrinolytic activity are known to induce the risk of thromboembolic complications in DCM patients.^37,38^ Insulin-like growth factor 2 (IGF2) and insulin-like growth factor-binding protein 2 (IGFBP2) were both highly up-regulated in DCM patients. These proteins have been observed to play an important role in the physiology and pathophysiology of cardiovascular diseases.^39^ Moreover, the mice overexpressing IGF2 evolved abnormalities in cardiac architecture, namely enlarged left ventricle.^40^

Taking into account the down-regulated proteins and annotation terms in DCM vs control, blood coagulation and lipid metabolism processes were the most prominent, which is in agreement with a previous proteomic study by Feig et al.^13^ Similarly, to our results, they observed a decreased level of several coagulation factors together with decreased apolipoproteins in DCM patients. Thromboembolic events as well as dysregulation in apolipoproteins and related cholesterol metabolism are serious complications in DCM and other cardiovascular diseases.

The most down-regulated protein in DCM vs control was titin (TTN), a large protein that occurs in a number of isoforms. There are various truncated TTN variants that belong to the most common genetic causes of DCM and PF4.^41,42^ The genetic variation of TTN in the studied cohort was not in the object of interest of the current study, and the used proteomic approach did not allow for specific variants discrimination. Nevertheless, the results show that in a subpopulation of the DCM patients, the canonical sequence of TTN was not detected. Serum paraoxonase/arylesterase 1 (PON1) and serum paraoxonase/lactonase 3 (PON3) were both similarly decreased in DCM patients. Both enzymes belong to the lipid metabolism pathways and low level of PON1 has been suggested a biomarker of DCM by Feig et al.^13^ Also the level of cartilage oligomeric matrix protein (COMP) was down-regulated in DCM patients similarly as in our previous study.^43^ Its reduced expression was observed in the heart tissues of DCM patients.^44^

We have noted also significant down-regulation of gelsolin (GSN), a known biomarker of rheumatic carditis,^45^ ficolin-3 (FCN3), which was also down-regulated in heart failure due to DCM or coronary heart disease,^46^ peptidase inhibitor 16 (PI16), known to be down-regulated in myocardial infarction^47^ and DCM,^41^ fibronectin (FN1), which is connected to coronary heart disease^48^ and DCM,^49^ or kallistatin (SERPINA4), which down-regulation in heart failure was connected to decreased survival time.^50^

To test the predictive value of DCM-associated proteins, we performed the logistic regression with elastic net optimization approach. The model trained on the measured samples with significantly deregulated proteins predicted the correct experimental outcome with 96% for DCM vs healthy control comparison. This result documents the great potential of identified DCM deregulated proteins as potential diagnostic biomarkers.

The comparison between LVRR– and LVRR+ DCM patient subgroups revealed a shorter list of significantly regulated proteins. The LVRR- samples were highly enriched in immunoglobulins and GO terms related to the immunoglobulin-mediated immune response. As discussed above, the immunoglobulins, their deposition in cardiomyocytes, and the overall malignant effect of autoimmunity on cardiac tissue are involved in the pathogenesis of idiopathic DCM.^27,29^ Immunoadsorption of selected autoantibodies has also been discussed as a potential therapeutic approach in DCM.^27^ Our results also suggest that higher plasma levels of immunoglobulins may be related to a worse response to common treatment strategies. Another annotation term that was up-regulated in LVRR- vs LVRR+ patients was the cell adhesion. Cell adhesion pathways have been found to be dysregulated on the transcriptome level in the DCM heart tissue.^51,52^

The most prominent among the proteins up-regulated in the nonresponding LVRR– patients was fructose-bisphosphate aldolase B (ALDOB). In a study by Rueda et al.^53^ ALDOB was used as one of four proteins to predict the low-term risk of mortality in patients with cardiogenic shock. Similarly, as in our study, the nonsurvivors of cardiogenic shock had significantly higher levels of ALDOB. We have also observed up-regulation of alanine aminotransferase 1 (ALT) in the LVRR– patients although the increase did not reach the level corresponding to severe liver damage. Higher level of this enzyme is one of the parameters of liver function abnormalities that are frequently observed in patients with heart failure,^54^ and its prognostic potential in heart failure has been also studied.^55^ Interleukin-1 receptor accessory protein (IL1RAP) was elevated in LVRR- patients. According to Niazy et al.,^56^ plasma and myocardial levels of IL-1 and its receptors increase in heart failure and remain increased upon implantation of MCS devices.

Three proteins that are part of ferroptosis pathway were here found to be up-regulated in LVRR- patients: transferrin receptor protein 1 (TFRC), serotransferrin (TF) and ceruloplasmin (CP). Ferroptosis belongs to nonapoptotic regulatory cell death mechanisms and its up-regulation is associated with many types of cardiomyopathies,^52^ especially the diabetic cardiomyopathy.^57^ The only significantly down-regulated protein in LVRR- patients was lipopolysaccharide-binding protein (LBP). LBP recognizes and binds bacterial lipopolysaccharides (LPS) and is further involved in the immune response and LPS clearance from circulation. Lower LBP levels were found to be connected to higher cardiovascular disease risk in older adults.^58^

We have verified the prediction efficiency of the above-mentioned dysregulated proteins in the DCM patients stratified according to response to treatment, as well. In this case, we found the average 88% consensus with experimental data which still provides a promising predictive model capability.

### Limitations of the Current Study

There are few known limitations in the present study. The healthy control group that was used in the first comparison had slightly higher average age than the DCM patients’ group (59 vs 51 years), and they were not strictly fasting 12 h before the sampling as the DCM patients. Instead, the healthy controls followed these instructions: low-fat diet, no alcohol, and no heavy physical activity for 24 h before the blood collection. Further limitation lies in the lower number of patients in the second part of the study, where only those with the most severe initial state were included. On the one hand, such restriction enhances the differences between the LVRR+ and LVRR- groups, but on the other hand, the predictive power of the presented proteins could eventually be lower when less severe cases were included. Also, the logistic regression performed on a small number of samples leads to a lower reliability of the calculated model. Therefore, future validations of the obtained results on new and larger sample cohorts should be conducted, including a targeted proteomics approach on selected protein candidates.

## Conclusion

Using a proteomic approach, we revealed a number of proteins and functional biological terms that are dysregulated between DCM and healthy control plasma samples. Number of them were previously discussed as potential DCM biomarkers. The most DCM up-regulated terms corresponded to inflammatory response, wound healing, and complement cascade, which suggest that some subclinical inflammation processes may take part in the DCM pathogenesis either as a result of previously underwent infection and subsequent autoimmunity reaction or the inflammation may be the consequence of the DCM-induced tissue damage. Among the most relevant DCM up-regulated proteins were CRP, CD163, AOC3, PF4, IGF2, IGFBP2, and TNC. Down-regulation in DCM samples was observed in the biological processes of blood coagulation and lipid metabolism, both of which present risk factors in cardiovascular diseases. The most relevant down-regulated proteins in DCM were TTN, GSN, FCN3, PON1, PON3, and COMP. The most challenge of the present study was looking for proteins that distinguish DCM patients according to their treatment response. The protein with the best prognostic potential was ALDOB, and its elevated plasma level was found in patients with worse or fatal outcome. The patients with no or poor treatment response had also up-regulated immunoglobulins and pathways corresponding to immunoglobulin-mediated immune response, which also points to the importance of these processes in the DCM prognosis.

## Supporting Information Available

Supplementary Table 1: Complete list of all proteins identified and quantified in the comparison between healthy control and DCM samples along with details from the proteomic analysis (XLSX)
Supplementary Table 2: Complete list of all proteins identified and quantified in the comparison between LVRR– and LVRR+ DCM samples along with details from the proteomic analysis (XLSX)
Supplementary Table 3: Full list of enriched annotation terms from the DAVID analysis (XLSX)
Supplementary Figure 1. A violin plot of predictive model efficiency for 100 random sample selections used for logistic regression with elastic net optimization. Supplementary Figure 2. An overlay of ROC curves obtained from logistic regression with elastic net optimization for DCM vs healthy and LVRR+ vs LVRR- comparison (PDF)
Supplementary R-script: R-script containing commands for logistic regression with elastic net optimization using glmnet package in R (TXT)

## Abbreviations

DCM: dilatation cardiomyopathy
LVEF: left ventricular ejection fraction
LVRR: left ventricular reverse remodeling
LFQ: label-free quantification
ESC: European Society of Cardiology
ICD: implantable cardioverter-defibrillator
CRT: cardiac resynchronization therapy
MCS: mechanical circulatory support
GFR: glomerular filtration rate
ACEi: angiotensin converting enzyme inhibitor
ARNi: angiotensin receptor–neprilysin inhibitor
MRA: mineralocorticoid receptor antagonist
RT: room temperature
BCA: bicinchoninic acid
SDS-PAGE: sodium dodecyl sulfate–polyacrylamide gel electrophoresis
SDC: sodium deoxycholate
TFA: trifluoroacetic acid
MS: mass spectrometry
LC-MS/MS: liquid chromatography and tandem mass spectrometry analysis
ACN: acetonitrile
FDR: false discovery rate
GO: gene ontology
SD: standard deviation
PCA: principal component analysis
LPS: lipopolysaccharide
