# Supplementary material for: Proteomic Profiling of Dilated Cardiomyopathy Plasma Samples — Searching for Biomarkers with Potential to Predict the Outcome of Therapy
Source: J Proteome Res. 2024 Feb 16;23(3):971–84. doi: 10.1021/acs.jproteome.3c00691 (PMC10913098; doi:10.1021/acs.jproteome.3c00691)
Supplement: Supplementary file 4 — pr3c00691_si_004.pdf [file pr3c00691_si_004.pdf]

Proteomic Profiling of Dilated Cardiomyopathy Plasma Samples – Searching for Biomarkers with Potential to Predict the Outcome of Therapy

Jana Klimentova,[1,2] Pavel Rehulka,[1] Jiri Stulik,[1,3] Vera Vozandychova,[1,2] Helena Rehulkova,[1,2] Ivana Jurcova,[4] Marie Lazarova,[5] Renata Aiglova,[5] Jiri Dokoupil,[2] Juraj Hrecko,[2] and Radek Pudil[2]*

[1] University of Defence, Faculty of Military Health Sciences, Department of Molecular Pathology and Biology, Trebesska 1575, 50001 Hradec Kralove, Czech Republic

[2] The 1st Department of Internal Medicine – Cardioangiology, Medical Faculty of Charles University in Hradec Kralove and University Hospital Hradec Kralove, Sokolska 581, 50005 Hradec Kralove, Czech Republic

[3] Charles University, Faculty of Medicine in Hradec Kralove, Simkova 870, 50003 Hradec Kralove, Czech Republic

[4] Institute for Clinical and Experimental Medicine (IKEM), Videnska 1958/9, 14021 Prague, Czech Republic

[5] Department of Internal Medicine I – Cardiology, Faculty of Medicine and Dentistry, Palacky University and University Hospital Olomouc, Zdravotniku 248/7, 77900 Olomouc, Czech Republic

Corresponding author: jiri.stulik@unob.cz, Tel. +420 973 253 220

Supplementary Table 1. Complete list of all proteins identified and quantified in the comparison between healthy control and DCM samples along with details from the proteomic analysis.

Supplementary Table 2. Complete list of all proteins identified and quantified in the comparison between LVRR- and LVRR+ DCM samples along with details from the proteomic analysis.

Supplementary Table 3. Full list of enriched annotation terms from the DAVID analysis.

Supplementary R-script. R-script containing commands for logistic regression with elastic net optimization using glmnet package in R.

Supplementary Figure 1. A violin plot of predictive model efficiency for 100 random sample selections used for logistic regression with elastic net optimization.

Supplementary Figure 2. An overlay of ROC curves obtained from logistic regression with elastic net optimization for DCM vs. healthy and LVRR+ vs. LVRR- comparison.

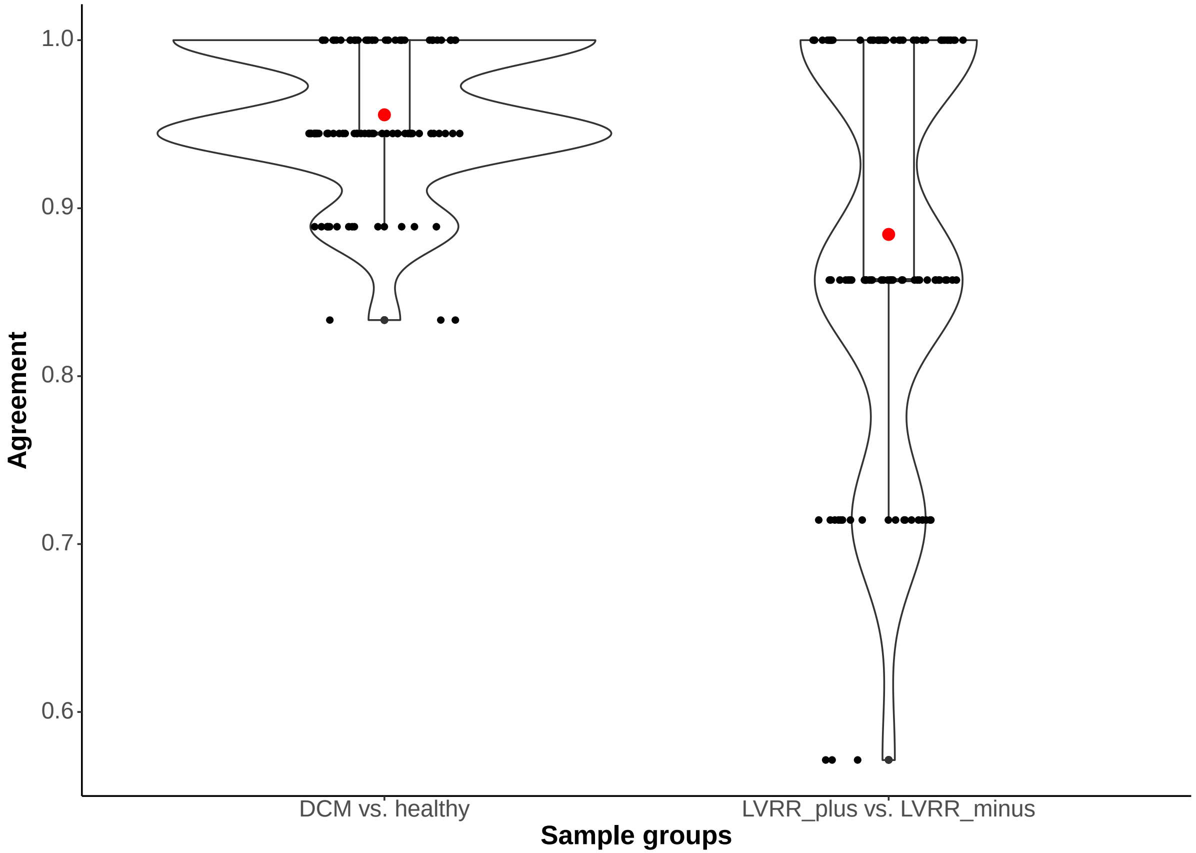

Supplementary Figure 1. A violin plot of predictive model efficiency for 100 random sample selections used for logistic regression with elastic net optimization. Left panel: results of modeling for the DCM patients vs. healthy control; right panel: results of modeling for the DCM patients stratified according to response to treatment.

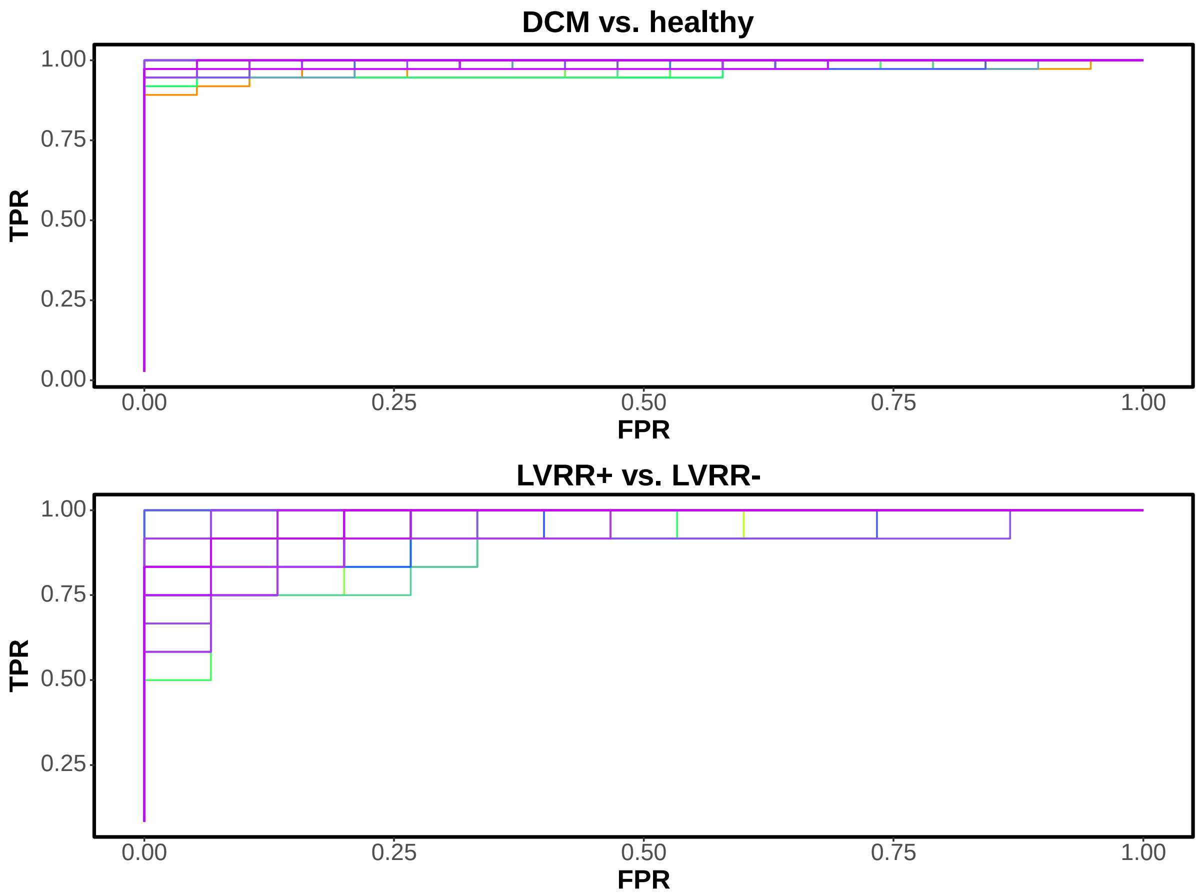

Supplementary Figure 2. An overlay of ROC curves obtained from multiple computation of logistic regression with elastic net optimization for DCM vs. healthy (top panel) and LVRR+ vs. LVRR- comparison (bottom panel). For each comparison, logistic regression with elastic net optimization was carried out for 100 random selection of training and testing data sets from obtained data. Different colors of ROC curves correspond to particular computation of the logistic regression.
